# Supplementary material for: The p55TNFR-IKK2-Ripk3 axis orchestrates arthritis by regulating death and inflammatory pathways in synovial fibroblasts
Source: Nat Commun. 2018 Feb 12;9:618. doi: 10.1038/s41467-018-02935-4 (PMC5809454; doi:10.1038/s41467-018-02935-4)
Supplement: Supplementary file 1 — Supplementary Information [file 41467_2018_2935_MOESM1_ESM.pdf]

Supplementary Figure 1

A

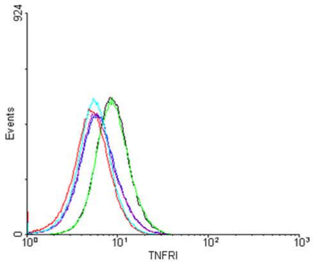

B

TNF$^{\Delta ARE}$ p55TNFR$^{f/f}$ ***TNF$^{\Delta ARE}$ p55TNFR$^{Ms\text{-}KO}$***

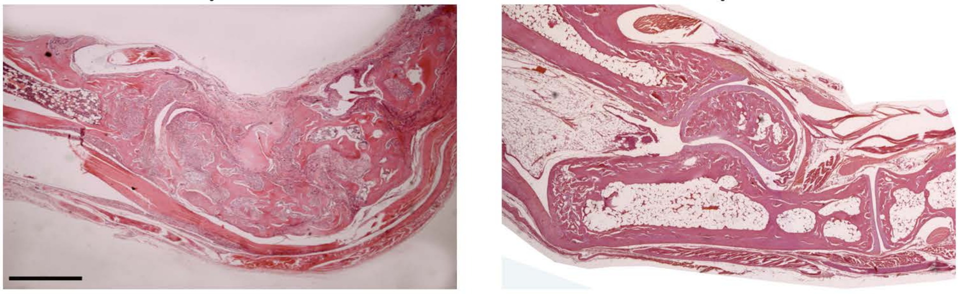

Mesenchymal p55TNFR-mediated signals are necessary for TNF-mediated arthritis

A. Flow cytometric analysis of p55TNFR (TNFRI) levels in SF cultures derived from *p55TNFR$^{Ms\text{-}KO}$ (light and dark blue lines)*, p55TNFR$^{\Delta/\Delta}$ (red line) and control *p55TNFR$^{f/f}$ (green line) and WT (black line)* mice. B. Representative histological images of H&E stained ankle joint sections of *TNF$^{\Delta ARE}$ p55TNFR$^{f/f}$* and *TNF$^{\Delta ARE}$ p55TNFR$^{Ms\text{-}KO}$*(age 56 weeks) Scale bars: 1mm. Total mice examined n=20-28 mice per genotype -age between 12 and 56 weeks of age).

Supplementary Figure 2

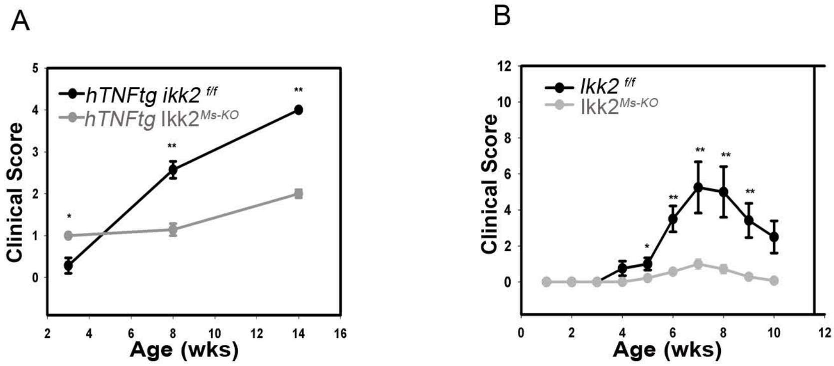

Mesenchymal IKK2-mediated signals are necessary for the development of TNF-mediated arthritis

A. Clinical scoring of arthritic manifestations of *hTNFtg Ikk2*$^{f/f}$ (n=6) and *hTNFtg Ikk2*$^{Ms\text{-}KO}$ (n=8) animals. B. Representative clinical scoring of arthritic manifestations of *Ikk2*$^{f/f}$ and *Ikk2*$^{Ms\text{-}KO}$ animals subjected to CAIA. (Total 3 experiments, n=20-22 per genotype). Data are presented as the mean ± SEM. *P < 0.05 and **P < 0.01, and ***P < 0.001, by 2-tailed Student's t test.

Supplementary Figure 3

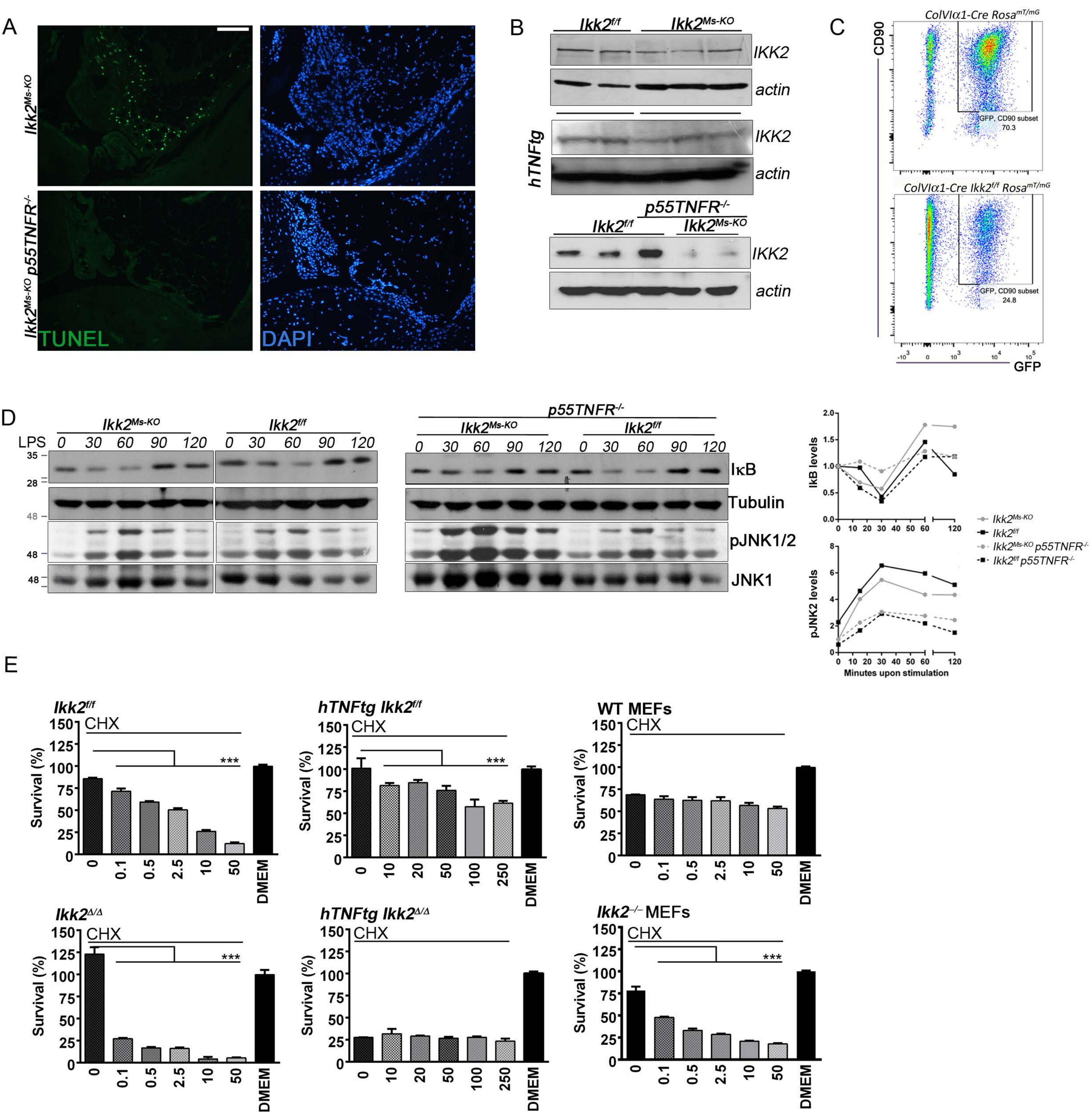

IKK2 signals regulate SF survival in vivo and ex vivo.

A. Representative TUNEL staining in ankle joint sections of *Ikk2*$^{Ms\text{-}KO}$ and *Ikk2*$^{Ms\text{-}KO}$ *p55TNFR*$^{-/-}$ mice (n=4 per genotype). B. Representative immunodetection of IKK2 in SF cultures derived from *Ikk2*$^{f/f}$ and *Ikk2*$^{Ms\text{-}KO}$ mice (naïve or hTNFtg) and *Ikk2*$^{Ms\text{-}KO}$ *p55TNFR*$^{-/-}$ mice. C. Representative flow cytometric analysis of SF cultures derived from *ColVIα1-Cre mT/mG* and *ColVIα1-Cre Ikk2*$^{f/f}$ *mT/mG* mice exhibiting the recombination efficiency of Cre in SFs in indicated genotypes (n=3-5): Mean values (±SEM) for % GFP;CD90+ cells: 66.3%±1.7 and 24.7%±0.5 for *ColVIα1-Cre Rosa*$^{mT/mG}$ and *ColVIα1-Cre Ikk2*$^{f/f}$ *Rosa*$^{mT/mG}$ SFs respectively). D. LPS-mediated IkBα degradation and JNK1/2 phosphorylation in *Ikk2*$^{f/f}$ and *Ikk2*$^{Ms\text{-}KO}$ (cropped photo derived from the same gel) and *Ikk2*$^{f/f}$ *p55TNFR*$^{-/-}$ and *Ikk2*$^{Ms\text{-}KO}$*p55TNFR*$^{-/-}$ SF cultures. E Representative survival rates of *Ikk2*$^{Δ/Δ}$ and *hTNFtg Ikk2*$^{Δ/Δ}$ SFs and *Ikk2*$^{-/-}$ MEFs and control *Ikk2*$^{f/f}$ and *hTNFtg Ikk2*$^{f/f}$ SFs and WT MEFs, treated with different TNF concentrations in the presence of 0.5ug/ml CHX for 18h (n= 15, 4 experiments). Data are presented as the mean ± SEM. *P < 0.05 and **P < 0.01, and ***P < 0.001, by one-way ANOVA (E). Unprocessed original scans of blots are shown in Supplementary Fig. 8.

Supplementary Figure 4

A

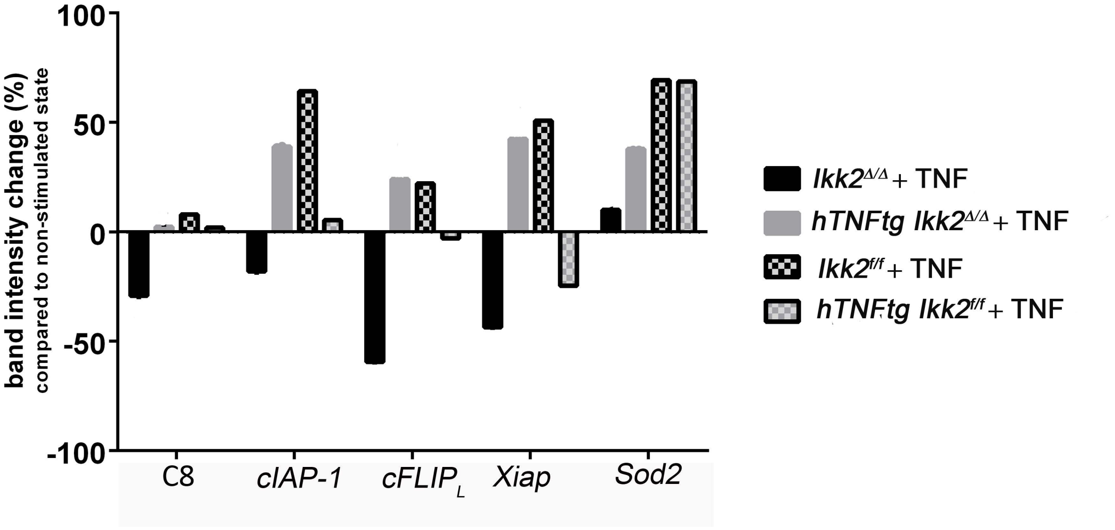

B

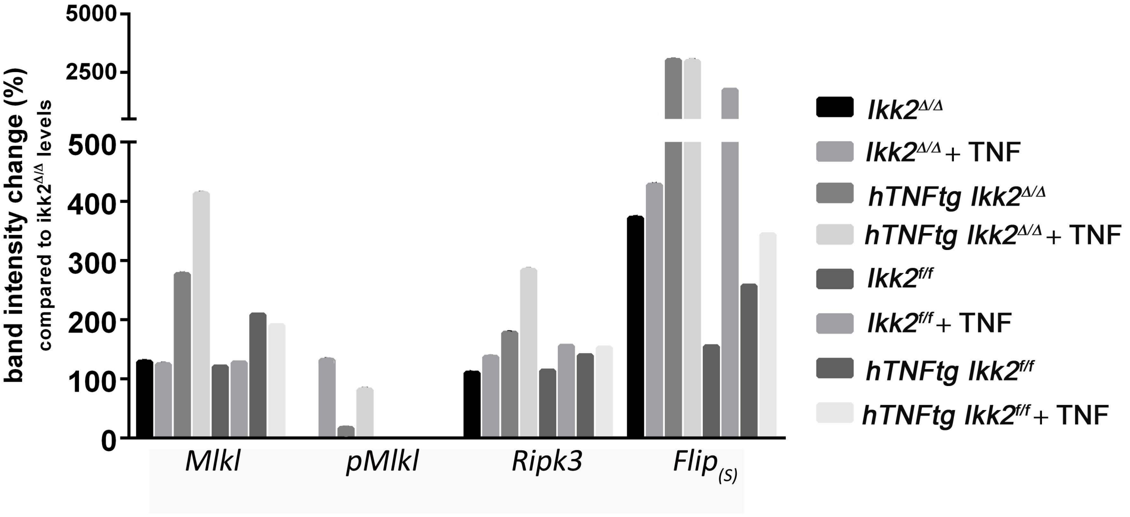

Quantitations of Western blots (Relative to Figure 4)

A. Relative quantitation of C8, cIAP-1, $cFLIP_{(L)}$, Xiap and Sod2 levels in TNF-stimulated SFs of indicated genotypes compared to the respective naive levels of each genotype (Fig 4C). B. Relative quantitation of Mlkl, pMlkl, Ripk3 and $cFLIP_{(S)}$ levels compared to *ikk2*$^{\Delta/\Delta}$ levels (Fig. 4F).

Supplementary Figure 5

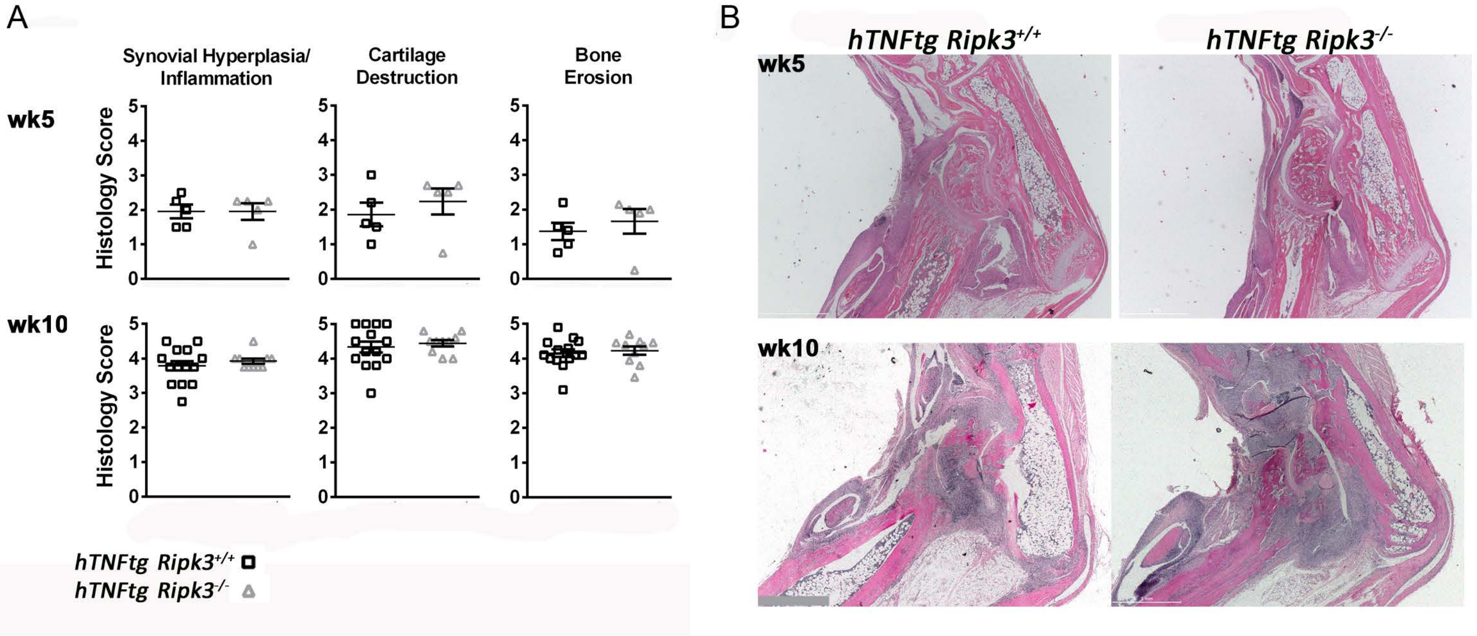

Ripk3 deficiency does not contribute to arthritic pathology of *hTNFtg* mice.
A and B. Histological score of synovial hyperplasia, cartilage destruction and bone erosion, and representative histological images of H&E stained ankle joint sections of *hTNFtg Ripk3*$^{-/-}$ and control-littermate *hTNFtg Ripk3*$^{+/+}$ mice. Scale bar: 1mm. Data are presented as the mean ± SEM. *P < 0.05 and **P < 0.01, and ***P < 0.001, by 2-tailed Student's t test.

Supplementary Figure 6

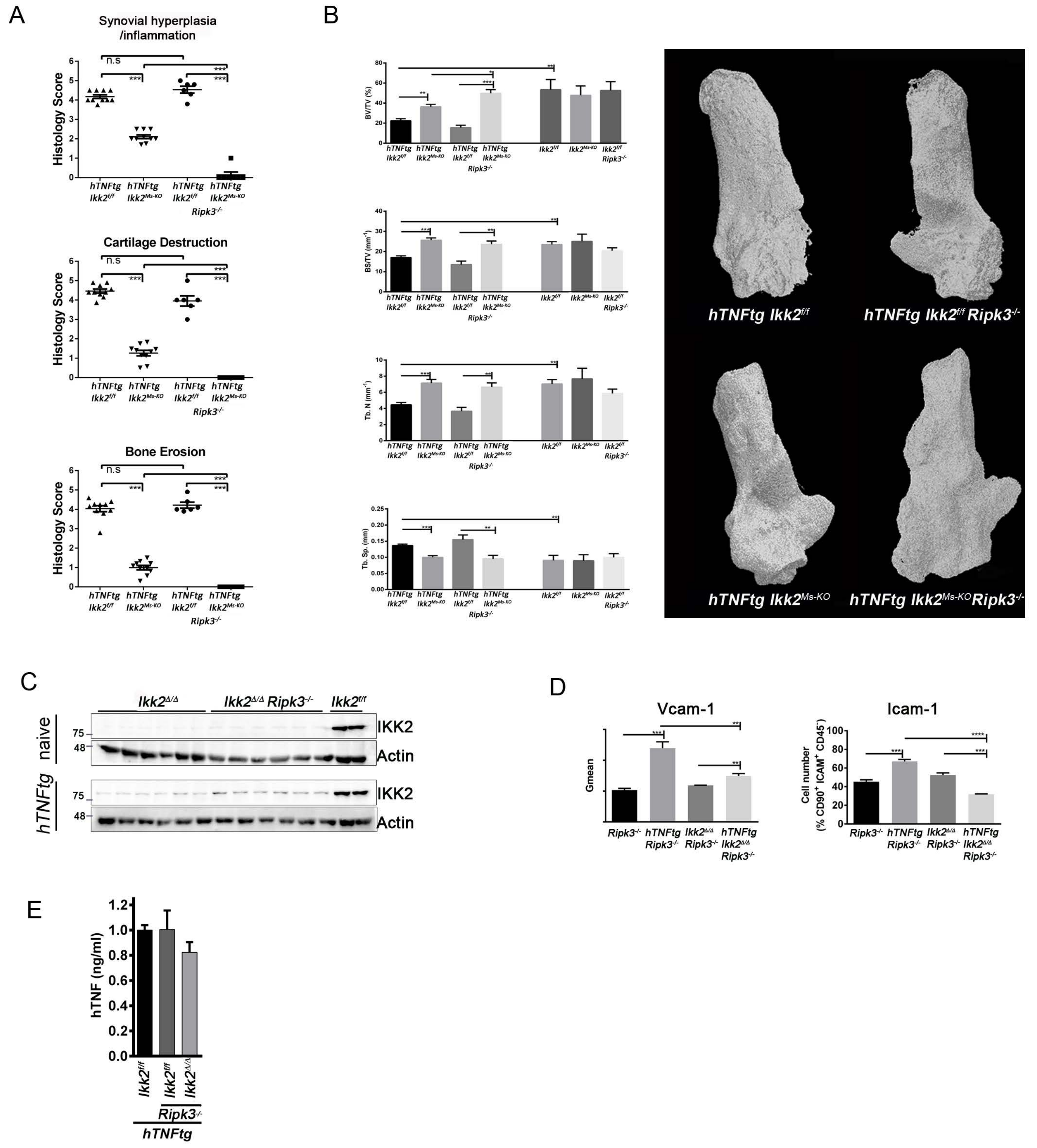

Ripk3 deficiency neutralizes the persisting synovitis observed in *hTNFtg* Ikk2$^{Ms\text{-}KO}$ mice

A. Histological score of synovial hyperplasia, cartilage destruction and bone erosion of ankle joint sections of indicated genotypes (week11) B. Effects of MS IKK2 and Ripk3 deficiency on the trabecular bone volume fraction of the distal calcaneus metaphysis in hTNFtg background and littermate controls. Using a 3-D image analysis system the bone volume fraction (BV/TV), bone surface density (BS/TV), the Trabelular number (Tb.N) and separation (Tb.Sp) was calcucated (n=4-8 mice per genotype, week11). Note the similarities in values among the non-*hTNFtg* groups, the differences when the *hTNFtg* background is introduced in the Cre^{-} mice and the shift of values when Cre expression is introduced in the *hTNFtg* mice (Left panel). Representative 3D-reconstructed images of the calcaneus from indicated genotypes (Right panel). C. Immunodetection of IKK2 after application of TAT-Cre in *Ikk2*$^{f/f}$, *Ikk2*$^{f/f}$ *Ripk3*$^{-/-}$ SF cultures in both naïve and hTNFtg background (marked as *Ikk2*$^{\Delta/\Delta}$ and *Ikk2*$^{\Delta/\Delta}$ *Ripk3*$^{-/-}$ respectively). D. Representative quantitation of flow cytometric analysis of Vcam-1 and Icam-1 levels in *Ikk2*$^{\Delta/\Delta}$ *Ripk3*$^{-/-}$ and *hTNFtg Ikk2*$^{\Delta/\Delta}$ *Ripk3*$^{-/-}$ SF cultures compared to *Ripk3*$^{-/-}$ and *hTNFtg Ripk3*$^{-/-}$ control cultures (n=4-5, 2 experiments). E. hTNF quantitation in supernatants of control *hTNFtg Ikk2*$^{f/f}$ (black column), *hTNFtg Ikk2*$^{f/f}$*Ripk3*$^{-/-}$ (dark grey column) and *hTNFtg Ikk2*$^{\Delta/\Delta}$*Ripk3*$^{-/-}$ (grey column) SF cultures (n=4-5, 2 experiments). Data are presented as the mean ± SEM. *P < 0.05 and **P < 0.01, and ***P < 0.001, by 2-tailed Student's t test. Unprocessed original scans of blots are shown in Supplementary Fig. 8.

Supplementary Figure 7

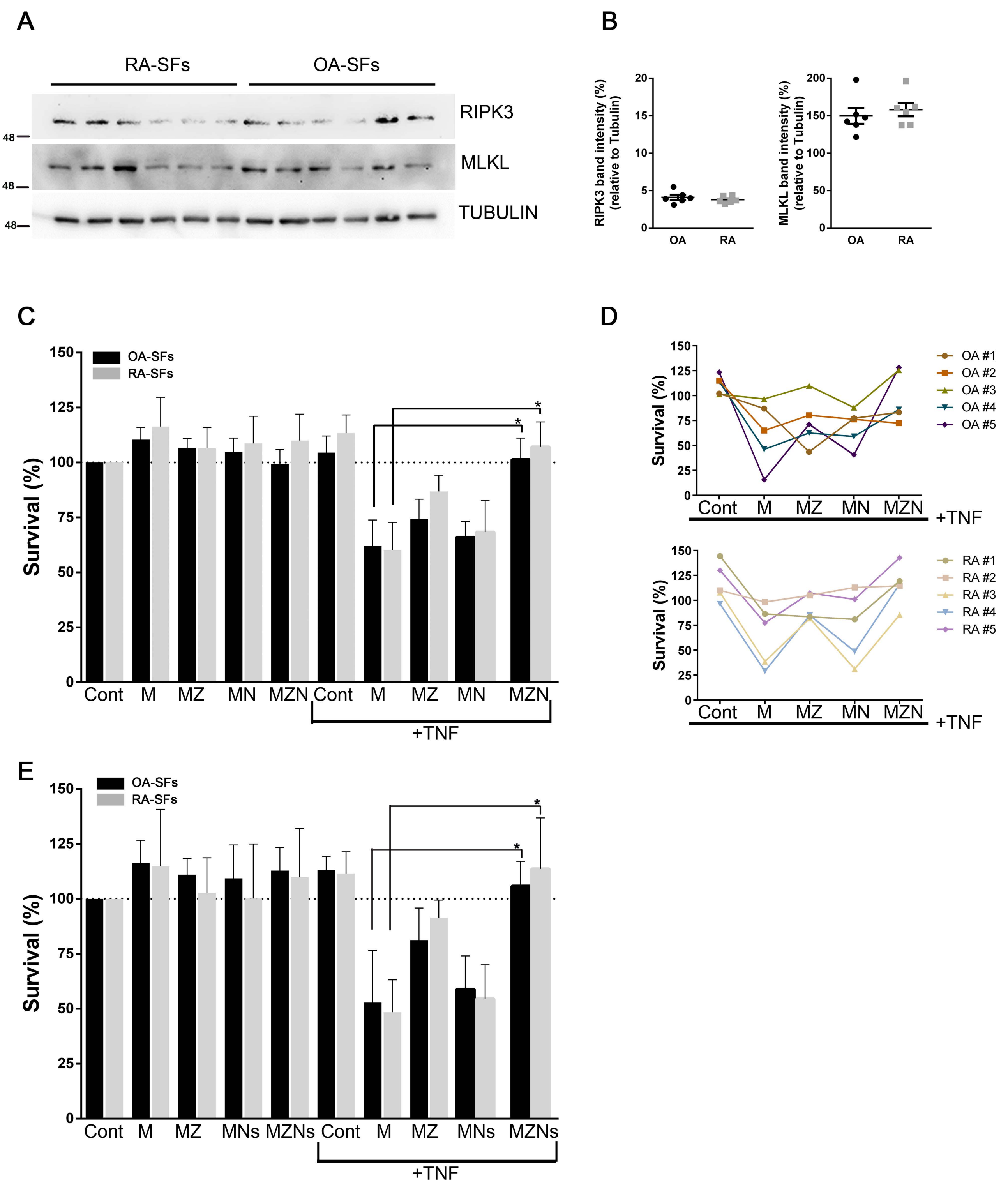

OA- and RA-SFs are prone to RIPK1-mediated, caspase -dependent and -independent death entities

A. Immunodetection of RIPK3 and MLKL levels in SF cultures derived from 6 RA- and OA-SF cultures. B. Quantitation of RIPK3 and MLKL levels relative to tubulin expression levels C. Survival rates of RA- and OA-SF cultures in response to different treatments in the absence or presence of TNF-10ng/ml (Cont: DMSO; M:MG132-50uM, Z: zVAD-20uM, N: Nec1-30uM) (n=5 per group). D. Survival rates of individual samples (depicted in (C))upon different treatments in the presence of TNF. Note that samples #4 and #5 from either OA- (blue and purple line) and RA-SFs (light blue and light purple lines) are partially rescued by either zVAD or Nec1 treatments. E. Survival rates of RA- and OA-SF cultures in response to different treatments, including Nec1s (more specific Ripk1 inhibitor), in the absence or presence of TNF (Cont: DMSO; M:MG132, Z: zVAD, Ns: Nec1s-30uM) (n=3 per group). Data are presented as the mean ± SEM. $^{*}P < 0.05$ and $^{**}P < 0.01$, and $^{***}P < 0.001$, by 2-tailed Student's t test. The blots presented herein are the unprocessed original scans.

Supplementary Figure 8

Full unedited gel for Figure 3D

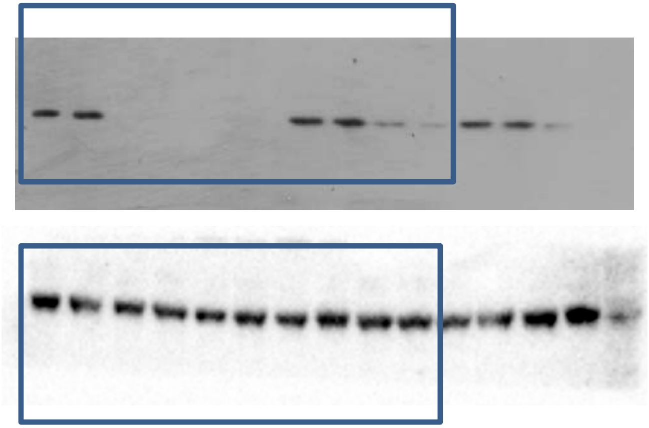

ikk2

tubulin

+TNF

+IL-1

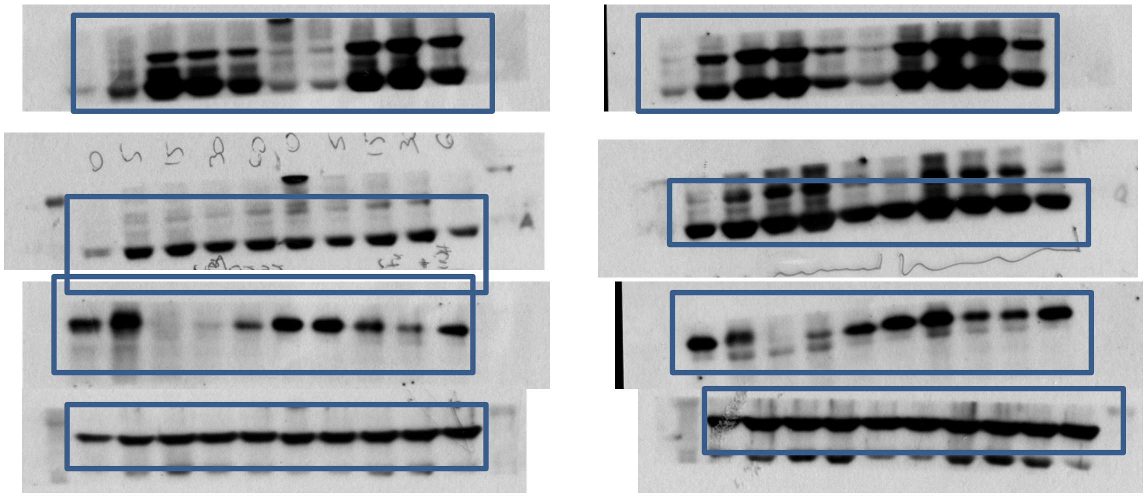

pJNK

JNK

ikb

tubulin

Supplementary Figure 8 (continued)

Full unedited gel for Figure 4A

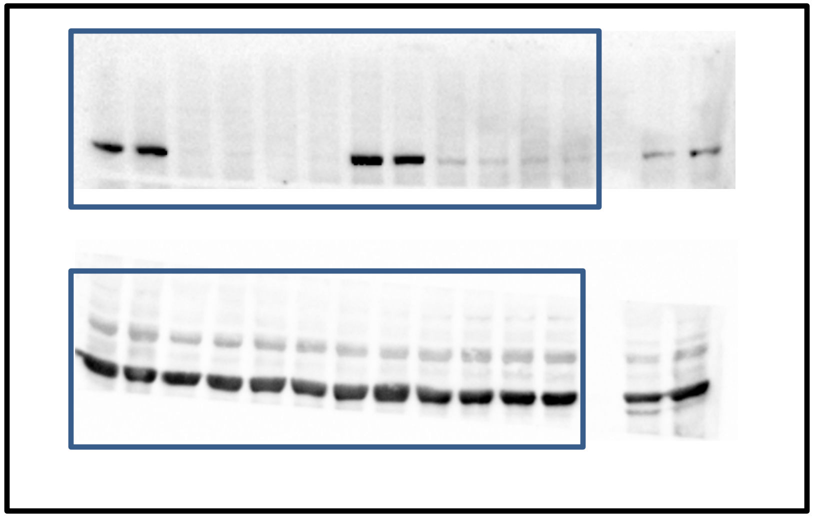

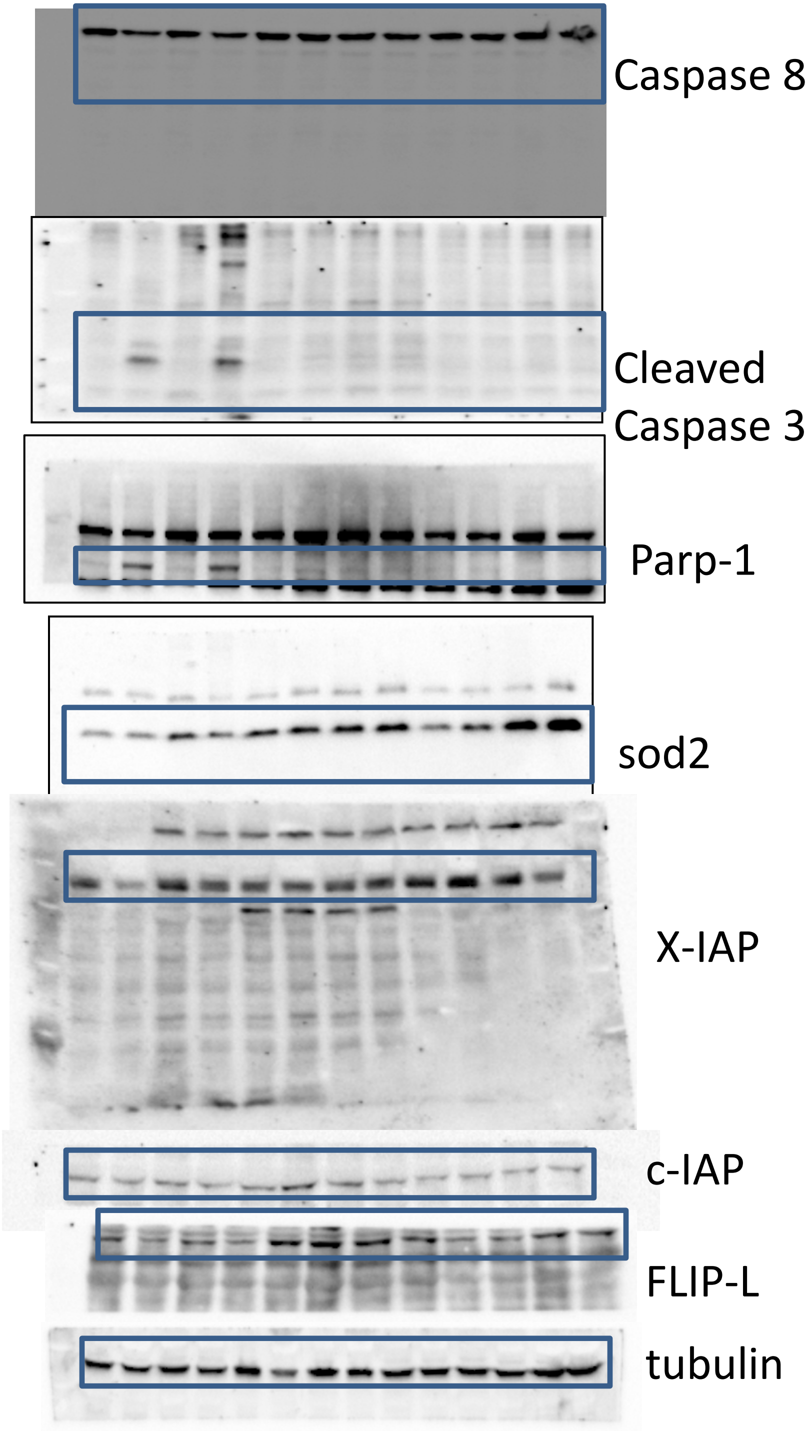

Supplementary Figure 8 (continued)

Full unedited gel for Figure 4e

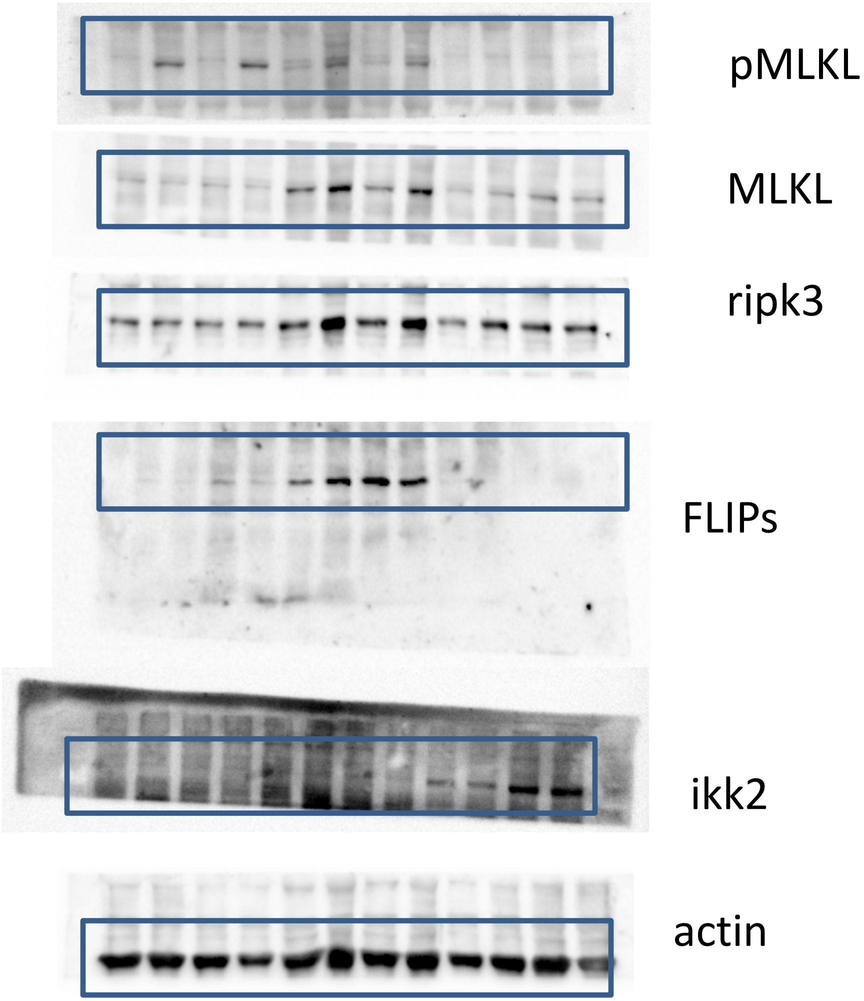

Supplementary Figure 8 (continued)

Full unedited gel for Figure 5c

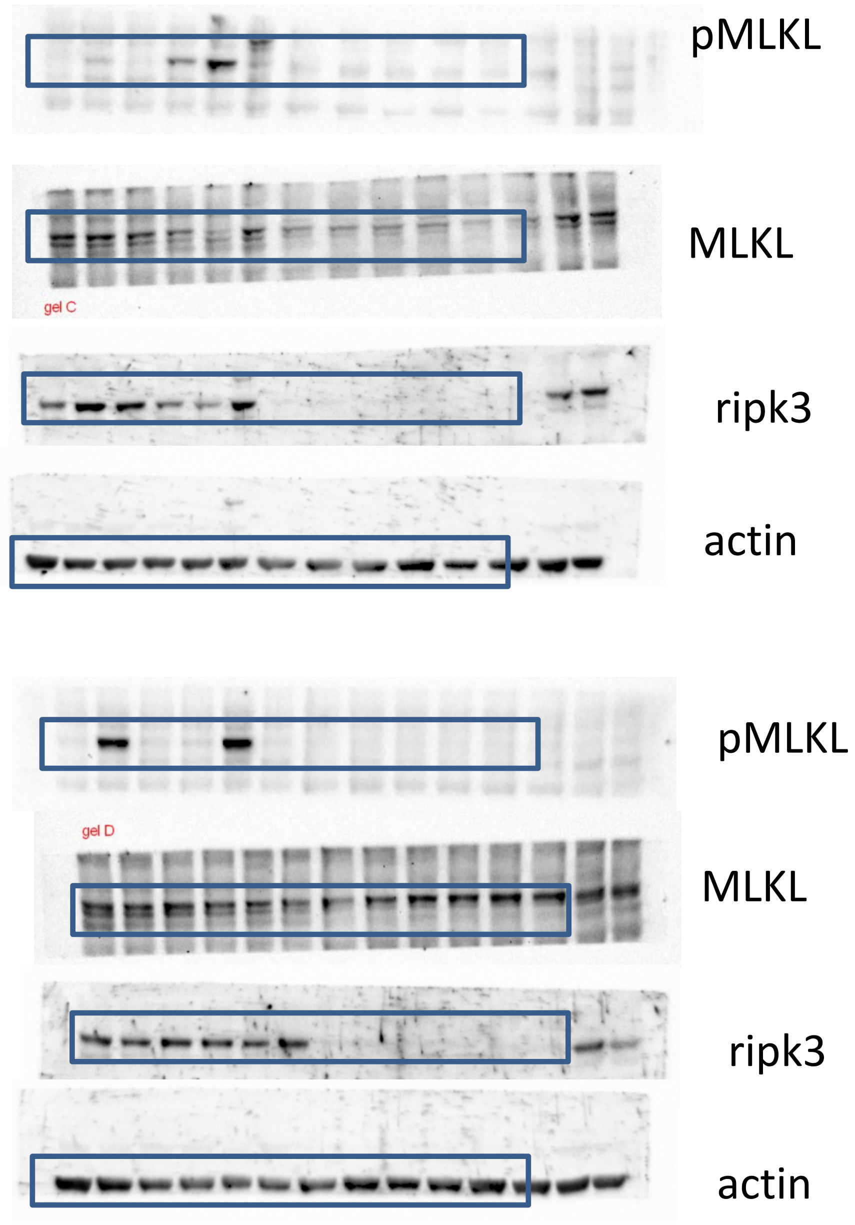

Full unedited gel for Figure 5d

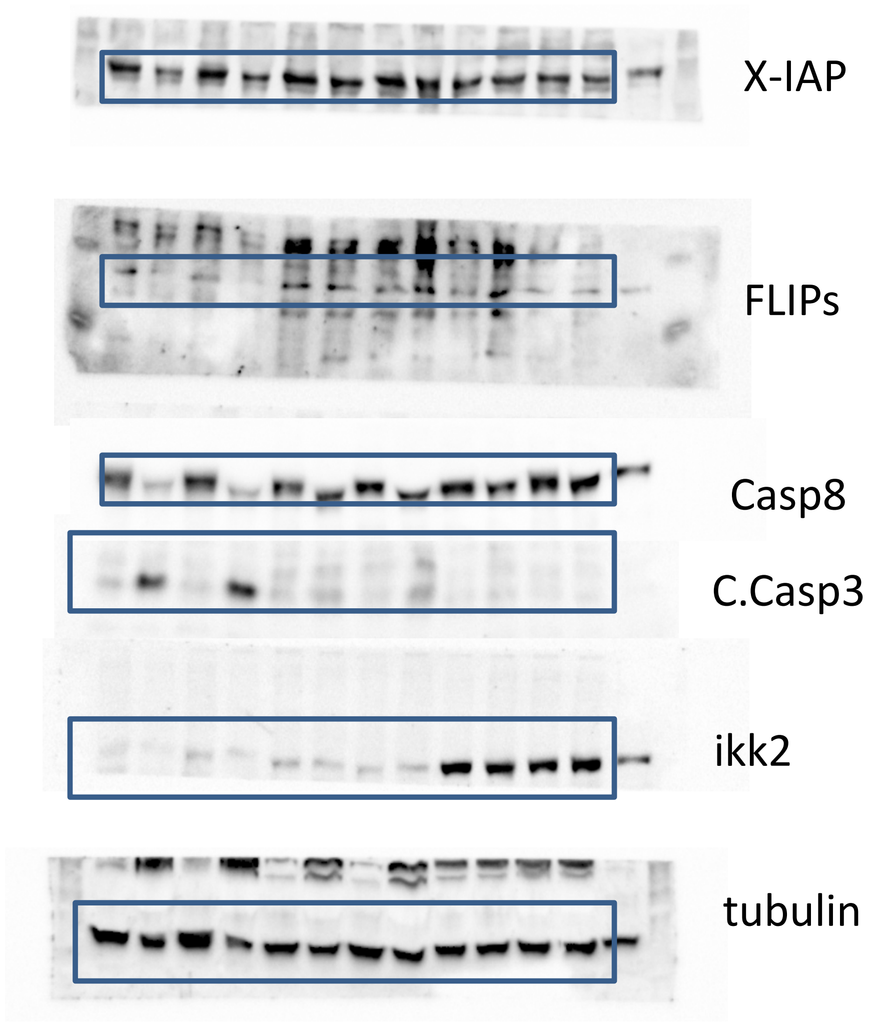

Supplementary Figure 8 (continued)

Full unedited gel for Supplemental Figure 3b

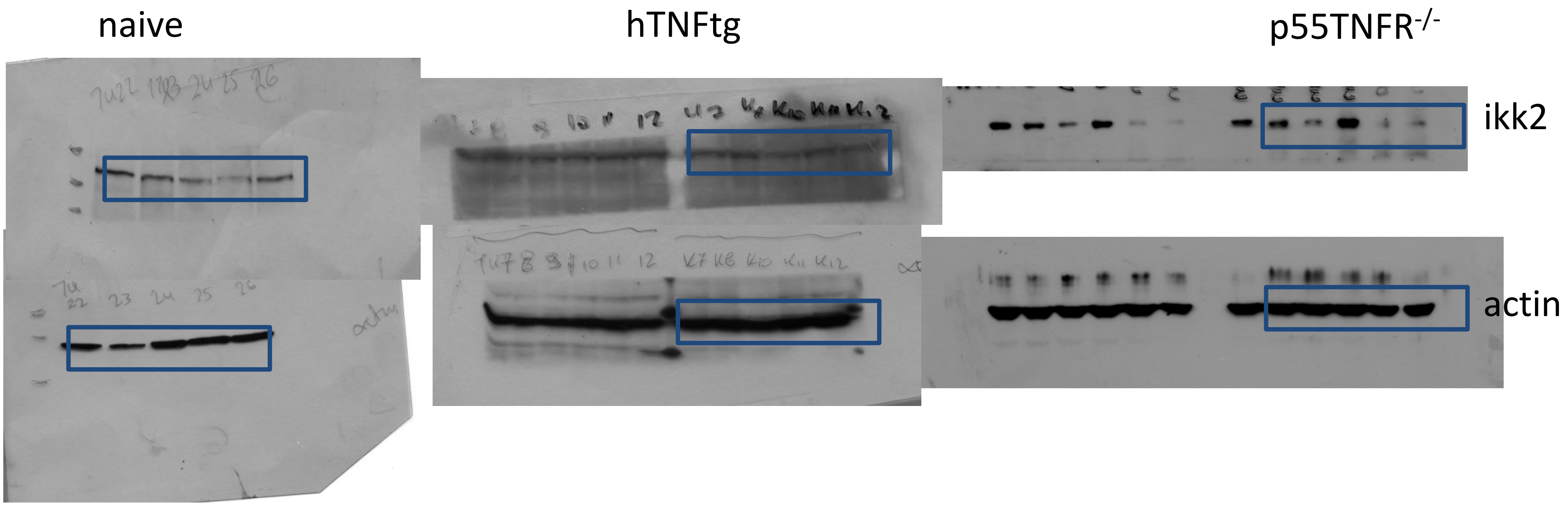

Supplementary Figure 8 (continued)

Full unedited gel for Supplemental Figure 3d

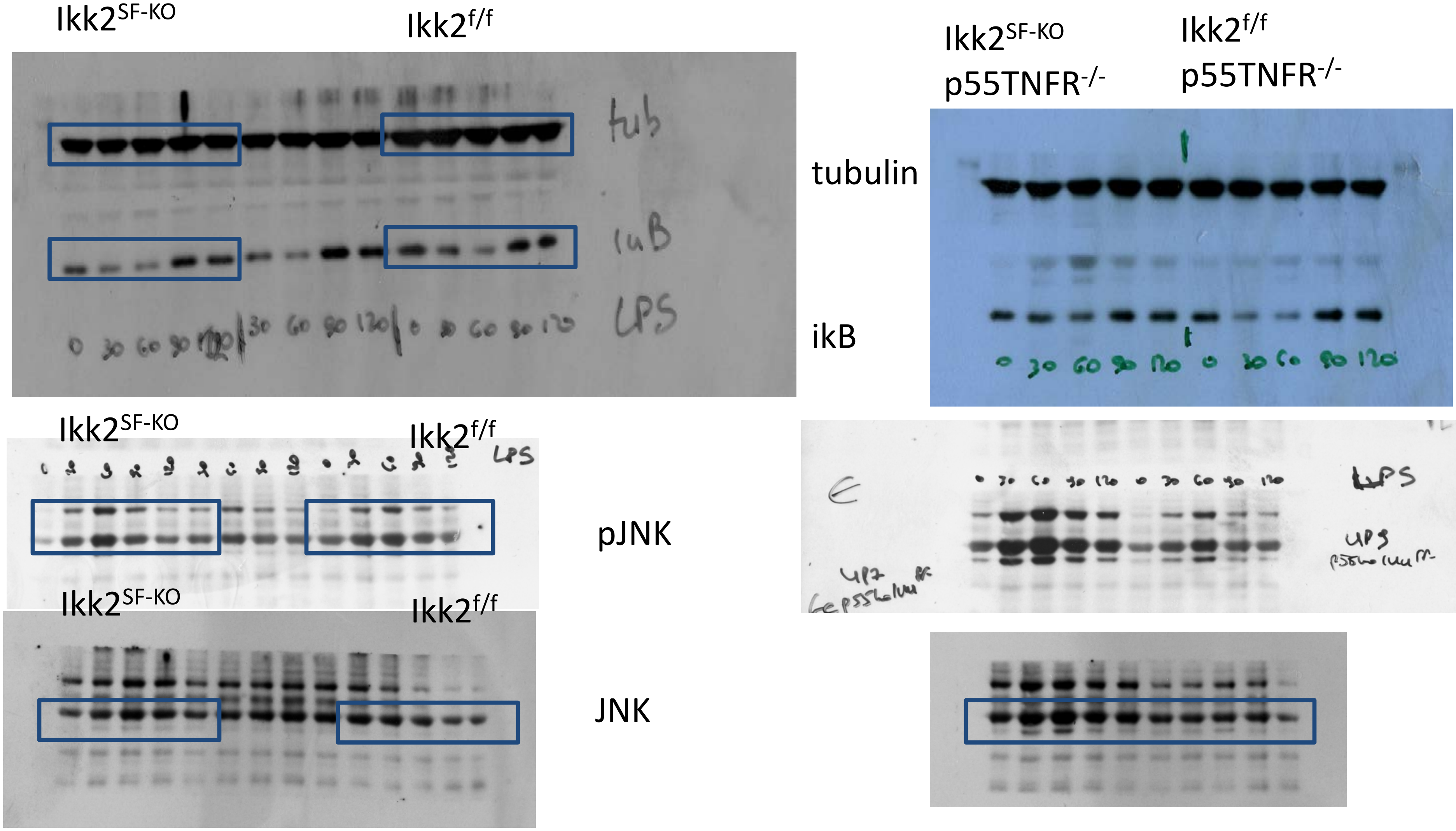

Supplementary Figure 8 (continued)

Full unedited gel for Supplemental Figure 4e

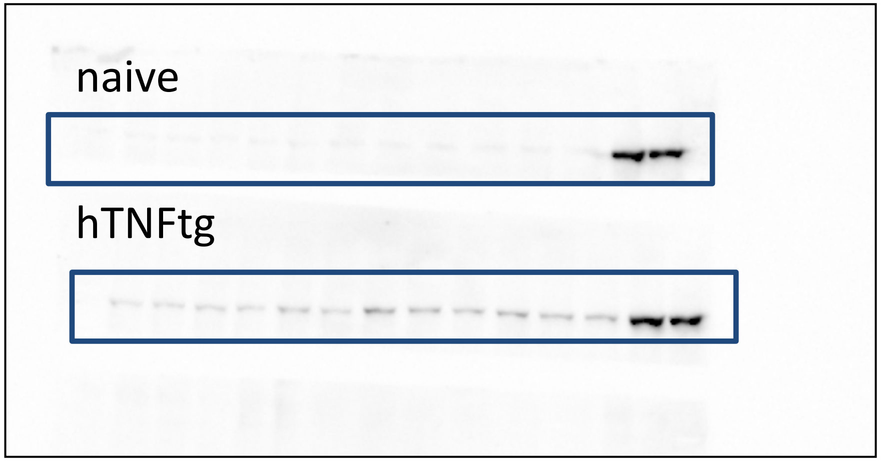

Ikk2
exposure

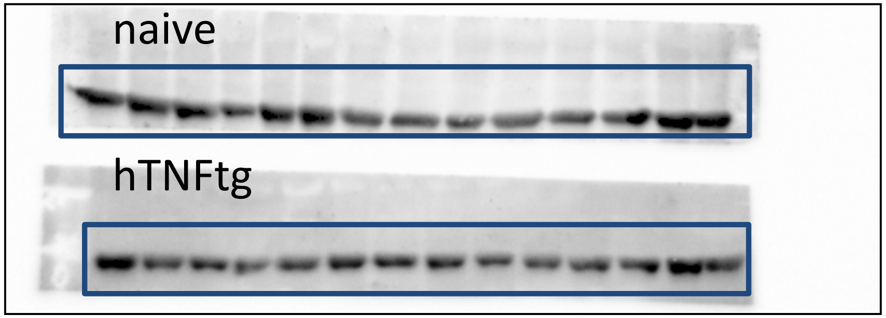

Actin
exposure

Supplementary Table 1. Histopathological criteria for scoring arthritic phenotype

score	Synovial Inflammation/ hyperplasia and pannus formation	Cartilage destruction	Bone erosion
0	Normal	Normal	Normal
1	Mild infiltration of inflammatory cells and/or mild edema	Minimal to mild, with no obvious chondrocyte loss	Small areas of resorption
2	Mild inflammation and hyperplasia	mild, with mild chondrocyte loss and proteoglycan disruption	More numerous areas of resorption
3	Moderate inflammation and pannus formation with superficial cartilage and bone destruction	moderate, with moderate multifocal chondrocyte loss and proteoglycan disruption	Obvious resorption of bone, without full-thickness defects in the cortex; loss of some trabeculae
4	Marked inflammation with pannus formation and moderate cartilage and bone destruction (depth to middle zone)	marked, with marked multifocal chondrocyte loss and proteoglycan disruption	Full-thickness defects in bone with partial distortion of the profile of remaining cortical surface
5	Severe inflammation with pannus formation and marked cartilage and bone destruction (depth to tidemark)	Severe diffuse, with severe multifocal chondrocyte loss and proteoglycan disruption	Full-thickness defects in bone with full distortion of the profile of remaining cortical surface

Supplementary Table 2. **Patient's characteristics**

Diagnosis	Joint	Passage	Sex	Age (years)	Disease duration (years)	Rheumatoid Factor	CRP (mg/l)	Treatment
RA	hand	5	F	45	19	positive	NA	MTX
RA	hand	5	F	55	18	positive	NA	corticosteroid
RA	hand	7	F	55	20	positive	24.4	corticosteroid, ciluzimab
RA	knee	7	M	61	9	positive	<10	MTX, infliximab, rticosteroid
RA	shoulder	6	F	66	20	positive	<10	rituximab
RA	shoulder	6	F	77	21	positive	<10	MTX, golimumab
RA	shoulder	7	F	53	21	positive	60.8	tofacitinib
RA	shoulder	5	F	56	12	positive	<10	MTX, infliximab
OA	hand	5	F	81	NA	NA	<10	none
OA	hand	6	F	61	NA	NA	<10	none
OA	hand	7	F	74	NA	NA	<10	none
OA	knee	6	F	59	NA	NA	<10	none
OA	knee	7	F	58	NA	NA	<10	none
OA	knee	6	F	72	NA	NA	<10	none
OA	shoulder	7	F	77	NA	NA	<10	none
OA	shoulder	6	F	79	NA	NA	<10	none
OA	shoulder	6	F	72	NA	NA	<10	none

Abbreviations: F = female, M = male, NA = not assessed, MTX = methotrexate
